# Antibiotic Sensitivity Profiling and Virulence Potential of *Campylobacter jejuni* Isolates from Estuarine Water in the Eastern Cape Province, South Africa

**DOI:** 10.3390/ijerph15050925

**Published:** 2018-05-06

**Authors:** Anthony C. Otigbu, Anna M. Clarke, Justine Fri, Emmanuel O. Akanbi, Henry A. Njom

**Affiliations:** Microbial Pathogenicity and Molecular Epidemiology Research Group (MPMERG), Department of Biochemistry and Microbiology, Department of Biochemistry & microbiology, University of Fort Hare, Private Bag X1314, Alice 5700, South Africa; aclarke@ufh.ac.za (A.M.C.); jfri@ufh.ac.za (J.F.); femo.emman@gmail.com (O.E.A); Hnjom@ufh.ac.za (H.A.N.)

**Keywords:** *Campylobacter jejuni*, physicochemical, virulence, drug resistance, estuary

## Abstract

*Campylobacter jejuni (CJ)* is a zoonotic microbe and a major causative organism of diarrheal infection in humans that often has its functional characteristics inactivated in stressed conditions. The current study assessed the correlation between recovered *CJ* and water quality parameters and the drug sensitivity patterns of the pathogen to frontline antibiotics in human and veterinary medicine. Water samples (*n* = 244) from rivers/estuarines were collected from April–September 2016, and physicochemical conditions were recorded on-site. *CJ* was isolated from the samples using standard microbiological methods and subjected to sensitivity testing to 10 antibiotics. Mean *CJ* counts were between 1 and 5 logs (CFU/mL). Ninety-five isolates confirmed as *CJ* by PCR showed varying rates of resistance. Sensitivity testing showed resistance to tetracycline (100%), azithromycin (92%), clindamycin (84.2%), clarithromycin and doxycycline (80%), ciprofloxacin (77.8%), vancomycin (70.5%), erythromycin (70%), metronidazole (36.8%) and nalidixic acid (30.5%). Virulence encoding genes were detected in the majority 80/95, 84.2%) of the confirmed isolates from *cdtB*; 60/95 (63.2%) from *cstII*; 49/95 (51.6%) from *cadF*; 45/95 (47.4%) from *clpP*; 30/95 (31.6%) from *htrB*, and 0/95 (0%) from *csrA*. A multiple resistance *cme*ABC active efflux pump system was present in 69/95 (72.6) isolates. The presence of *CJ* was positively correlated with temperature (*r* = 0.17), pH (*r* = 0.02), dissolved oxygen (*r* = 0.31), and turbidity (*r* = 0.23) but negatively correlated with salinity (*r* = −0.39) and conductivity (*r* = −0.28). The detection of multidrug resistant *CJ* strains from estuarine water and the differential gene expressions they possess indicates a potential hazard to humans. Moreover, the negative correlation between the presence of the pathogen and physicochemical parameters such as salinity indicates possible complementary expression of stress tolerance response mechanisms by wild-type *CJ* strains.

## 1. Introduction

*Campylobacter* spp. are of the epsilonproteobacteria class of microorganism [1]. They are slow growing, Gram-negative, spiral shaped, motile organisms, characterized by their microaerobic nature [2]. They have been reported to be detected in greater quantities in diarrhea infections in humans than any other enteric pathogen and they require less than 100 cells to infect a host [3]. Campylobacteriosis is a chronic enteric infection primarily caused by cytotoxin-producing *Campylobacters* that invade and colonize the gastrointestinal (GI) tract in humans [4]. It is a zoonotic disease mainly transmitted via the consumption of poultry products [2], contact with pets and livestock, ingestion of water contaminated with human faeces originating from sewage, septic tanks, latrines and even animal faeces or from raw milk [5]. In humans, the disease lasts between 4–7 days and is characterized by acute enteritis, fever, vomiting and abdominal pain [4], with the danger of possibly leading to some post-infectious neuropathic diseases, such as bacteraemia, Guillain–Barre syndrome (GBS), reactive arthritis (ReA), and abortion [4,6]. *Campylobacter jejuni* and *C. coli* are the two widely recognized pathogenic species of *Campylobacter* that cause diseases in humans [7]. A large number of recorded campylobacteriosis outbreaks have also been traced to the ingestion of untreated surface water contaminated through human activities or by avian wildlife faeces [8,9]. Recreational and potable water are potential reservoirs of *Campylobacter* infection [5]. Therefore, the role of water in the transmission of *Campylobacter* species is of significant importance.

### 1.1. Virulence Determinants

*Campylobacter jejuni* possesses some multiple cell surface expressive virulent factors responsible for its high prevalence and pathogenicity compared to other enteric bacteria [3,10]. The secreted toxin is either enterotoxic or cytotoxic in its mode of action [10]. The target sites of enterotoxins are the cells lining the gastrointestinal (GI) tract of the host where its virulent effect is felt, resulting in either cytotoxin-linked inflammatory diarrhea associated with fever or non-inflammatory diarrhea associated with enterotoxins, characterized by non-leukocyte watery stools [4].

#### 1.1.1. Cytolethal Distending Toxin (CDT)

The Cytolethal Distending Toxin (CDT) is an apoptosis triggering toxin produced by a group of Gram negative bacteria including *Campylobacter jejuni (CJ)* [9]. This toxin plays an important role in the host mucosal inflammatory response for interleukin-8 (IL-8) released by intestinal cells [10]. CDT is suggested to have an AB_2_ tripartite structure with *cdtB* as the main effector, while *cdtA* and *cdtC* are makeup units associated with cell membrane binding [11]. The *cdtA* protein has a molecular mass of 27 KDa, *Cdt*B has a molecular mass of 29 KDa, and *cdt*C has a molecular mass of 20 KDa [11]. Subunit A is the active unit directly responsible for DNA damage, while subunit B is a binding subunit that helps to bind the toxin to the specific target cells which inhibit cdc2, causing cellular distention and eventually death. The DNase activity of CDT is lethal—causing singular strand breakage with an estimated lethal dose (LD) of 50 pg/mL [11]. The pivotal role played by in cell and DNA degradation not only results in inflammatory diarrhea with faecal leukocytes but can potentially create lesions in fragmented DNA strands that can promote cancer [4,9]. CDT has high cross species sequence similarity and *cdt*B has the highest interspecies similarity [11]. It is, however, believed that some species lack *cdt*B but still have the potential to cause symptoms in children less than 3 years old [11].

#### 1.1.2. Campylobacter Invasion Antigens (*ciaB*)

This is a protein synthesized by *CJ* which facilitates invasion to epithelial cells [10] of the gastrointestinal tract where it inflicts increased damage on the columnar epithelial cells, leading to swelling and rounding of invaded cells as a result of the cytotoxin and enterotoxin activities [8,10]. *Campylobacter* heat labile cytotonic (CTON) and cytotoxin (CTOX) are associated with non-inflammatory and inflammatory diarrhea, respectively [11]. *Cia* proteins are suggested to modify host cell regulatory pathways to promote *CJ* pathogenicity [12].

#### 1.1.3. Fibronectin-Binding Protein (*cadF*)

This outer membrane conserved gene encodes a protein containing 326 amino acids of molar mass 37 kDa and plays a vital role in adherence to intestinal epithelial cells [13]. Internalization of the organism into its host is harnessed by the binding activity of *cadF* to the extracellular fibronectin [14]. However, studies have shown that there may be a reduction in *cadF* functionality when it assumes a defensive viable but non-culturable (VBNC) state [14].

#### 1.1.4. Sialyltransferases (*cstII*)

This is an outer core structure carbohydrate called lipo-oligosaccharide (LOS) expressed by *CJ* that evades detection by mimicry of the human gangliosides [15]. The mechanism of action of this gene is providing the LOS with a protective barrier which facilitates its invasion of the epithelial cells by portraying a resemblance to the human ganglioside in the vertebrate nerve cells, allowing the host’s immune system to self-destruct its own ganglioside [15]. It is believed to trigger the development of autoimmune diseases, such as Guillain–Barre Syndrome [16].

#### 1.1.5. Post Transcriptional Regulator (*csrA*)

*csr*A gene is a carbon starvation regulator gene linked to the encoding of protein regulation which plays a vital role in *CJ*’s ability to responsively regulate a stationary phase mechanism to withstand hostile conditions [17]. Other associated virulence expressed by this gene is related to oxidative stress survival, the adherence of intestinal epithelial cells and biofilm formation [18]. Biofilm formation is an adaptive mechanism which complements the fragility of an organism when exposed to stress conditions by triggering a switch into a VBNC state [19].

#### 1.1.6. ATP-Dependent Endopeptidase Protease (*clpP*)

This protease subunit in the bacterial caseinolytic proteases (CLP) contributes to virulence via energy formation through the degradation of virulence regulators [20], while indirectly playing a triggering role in stress tolerance of the organism when subjected to stress conditions [21]. The association of *clpP* with *clp* ATPase subunits enhances the proteolytic activity of the enzyme in the presence of ATP, producing a catalytic action [21]. In many pathogens, *clpP* enhances protein induced growth, under conditions, such as high temperature and oxidative stress [22].

#### 1.1.7. Periplasmic Chaperon (*htrB*)

This is a periplasmic chaperon gene that encodes an acyltransferase for lipid A synthesis [23,24]. Synthesis of this enzyme regulates the organism’s response to environmental changes [25]. It is, however, interesting to note that the *Campylobacter* species shows varying diversities of infection outcomes [26] attributable to differences in genetic composition [22].

### 1.2. Treatment and Drug Resistance

*Campylobacter* infection is, at times, regarded as self-limiting, but in the case of severe complications, antibiotics are commonly recommended, especially for immunodeficient patients. Antibiotic resistance among *Campylobacter* species has emerged as a global public health burden [4]. There are cases of growing resistance of *Campylobacter* spp. against the front line and alternative treatment therapies, such as macrolides (erythromycin), tetracycline, fluoroquinolones and aminoglycoside (gentamycin) [7,27]. The unregulated use of antimicrobial agents as food additives in livestock in order to prevent and control infections and enhance growth rates [28,29] has contributed to an increased resistance in microbes against multiple antibiotics [9]. The unregulated administration of fluoroquinolones to poultry has contributed to increased resistance of *CJ* to fluoroquinolones in industrialized regions [27].

A survey of the antimicrobial susceptibility of *Campylobacter* species isolated from poultry and pigs was carried out in the Western Cape and Gauteng provinces of South Africa and the results displayed clear traces of resistance to fluoroquinolones, macrolides and tetracycline antibiotics, while some of the isolates displayed multidrug resistance [30]. These characteristic drug resistances were prominent among two specific *Campylobacter* species, *CJ* and *C. coli*, which have very similar epidemiology, but require biochemical tests to distinguish between them [30]. Previous studies have reported variation in *CJ* sensitivity to erythromycin and ciprofloxacin; resistance rates of 79.2% were reported in Nigeria [31], 0% in Djibouti [14] and in Qatar, resistance rates of 63.2% to ciprofloxacin and 8.6% to erythromycin were shown [32]. Resistance to ciprofloxacin, another antimicrobial agent of consideration next to erythromycin, has also been recorded in some other parts of the world [33]. Multidrug resistance in *Campylobacter* is a widely studied area. Previous studies have suggested mutation as a factor responsible for the acquisition of this characteristic [34,35,36]. *Campylobacters* have an innate resistance trait in combination with externally acquired resistance traits to express virulence [35]. Mutation is believed to play a role in the evolution of the *cme*ABC operon [15,36] in the multidrug efflux system. Drug resistance has, however, been attributed to target modification-mediated enzymatic inactivation and enhanced efflux [37].

## 2. Materials and Methods

### 2.1. Study Area

The Swartkops estuary (33°52′ S; 25°38′ E) was selected for this study, and it is one of the most important estuaries in South Africa. It is also an important bird area (IBA) harbouring approximately 4000 migratory birds annually [38]. It is located close to the coastal city of Port Elizabeth in Nelson Mandela Bay Municipality of the Eastern Cape Province. The river is approximately 134 km long, while the estuary is approximately 16.4 km long with a permanent open connection into Algoa Bay in the Indian Ocean [39]. The total catchment area of the Swartkops River (including the tributary) is about 1360 km^2^ [39]. Surrounding areas in the catchment of the Swartkops River are used for agriculture, while the lower reaches of the river and the estuary are surrounded by extensive human development, including several industries [40].

### 2.2. Sampling and Isolation of CJ

#### Sampling and Isolation

**Figure 1:**
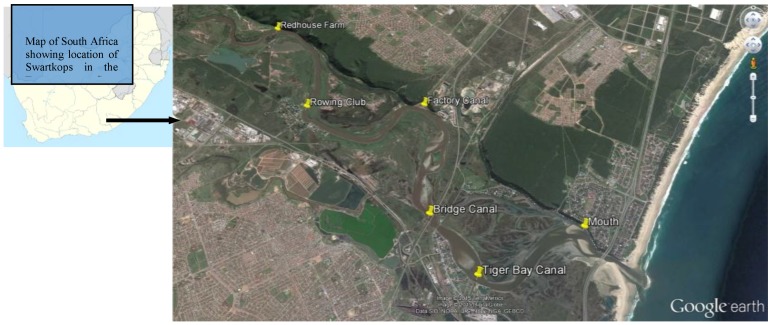
Location and GPS coordinates of the study sites in the Swartkops River estuary, Port Elizabeth. Sampling locations within the estuary; Rowing Club (RC) (33°50’14.60 S; 25°34’14.37 E); Factory Dam (FD) (33°50’12.16 S; 25°35’41.24 E); Redhouse Farm (RF) (33°49’10.56 S; 25°33’37.44 E); Bridge Canal (BC) (33°51’25.99 S; 25°35’49.99 E); Despatch Mouth (DM) (33°51’32.22 S; 25°37’31.33 E); Tiger Bay Canal (TB) (33°52’0.26 S; 25°36’22.32 E) (https://www.google.com.au/maps/).

The spot sampling method, as described by the JEEP92 project [41], was used. An Aestuaria Bandi 410 vessel was used for sampling a total distance of 12.775 km of the Swartkops river estuary between the six sample points (Figure 1). Triplicate water samples were collected against water flow from surface level and at a depth below (3 m) using sterile bottles from each sampling point over a 6-month period (April–September 2016) covering three seasons (autumn, winter and spring) of the year and transported at 4 °C to the laboratory and analyzed within 5 h after collection. Physicochemical parameters (temperature, pH, electrical conductivity, salinity and turbidity) of sample stations were recorded in-situ [42] using the YSI 650 MDS multi-parameter reader at two levels (surface and bottom) from each sampling point.

Bacteria cells were concentrated on a microfilter (0.65 µm pore size cellulose ester Millipore) from raw water (100 mL; 10^−1^; 10^−2^) samples. The concentrated filter was aseptically folded and enriched in 20 mL nutrient broth supplemented with Preston *Campylobacter* selective supplement (SR0117-Oxoid) with 5% lysed horse blood and incubated microaerobically at 37 °C for 48 h. One hundred microlitres of enrichment culture was sub-cultured to *Campylobacter* blood-free agar (CCDA; CM739; Oxoid) containing CCDA selective supplement (SR155E; Oxoid) and incubated microaerobically using a campy gas pack (5% O_2,_ 10% CO_2_, 85% N_2_, CampyGen Oxoid) at 37 °C for 72 h. All plating was carried out in duplicate. Distinct presumptive colonies on each plate were counted by the surface count method to determine the total viable *Campylobacter* counts (TVCC). Then, 8–10 different colonies per plate were picked and subcultured on the selective medium for purity. A positive control (*Campylobacter jejuni* ATCC 33560 strain) was included with each set of tests. Identification of positive isolates was based on colony morphology, Gram-stain, no-growth in aerobic condition, hippurate hydrolysis and oxidase tests.

### 2.3. PCR Confirmation of CJ and Detection of Virulence Genes

Genus confirmation was performed using the 23S rRNA gene [43], and species confirmation was done using the *hipO* gene [44]. Screening for the pathogenic virulence genes, *cdtB*, *cadF*, *cstII*, *csr*A, *htrB* and *clpP*, was performed on the *CJ* confirmed isolates. Genomic isolation from the confirmed isolates was carried out using a commercial genomic DNA isolation kit (Qiagen Kit, Invitrogen, Thermo Fisher Scientific, USA) according to the manufacturer’s instructions. Table 1 shows the primer sequences (Inqaba biotech, South Africa), amplicon sizes and cycling conditions of the various genes used in this study. The final concentration of the 25 µL PCR reaction consisted of 12.5 µL of the 2X master mix (Sybrselect, USA) 0.5 µL forward and reverse primers, 6.5 µL molecular grade water and 5 µL template DNA. The cycling conditions were as follows: initial denaturation was at 94 °C for 5 min, and then 94 °C for 30 s, with modifications in annealing temperatures specific to the primer pair (as given in Table 1) for 5 min and extension at 72 °C for 50 s. All PCR products were analyzed by electrophoresis on 1.5% agarose gels (CSL-AG100, Cleaver Scientific Ltd. Warwickshire, UK) except for *htrB* and *clpP* genes which were analyzed on 2% agarose gels. The gels were stained with ethidium bromide and visualized with a UV transilluminator and photographed (Alliance 4.7).

### 2.4. Antimicrobial Sensitivity Testing

Confirmed *CJ* isolates were subjected to antimicrobial sensitivity testing with 10 antimicrobial agents. The Kirby–Bauer disk diffusion method on Mueller–Hinton agar supplemented with 5% horse blood, in accordance with Clinical and Laboratory Standards Institute guidelines [38] was used to perform the antimicrobial profiling. An inoculum of each bacterial isolate was emulsified in 3 mL of sterile normal saline (0.9%) in test tubes and the density was adjusted to 0.5 McFarland standard (0.5 mL of 1% *w*/*v* BaCl_2_ and 99.5 mL of 1% *v*/*v* H_2_SO_4_), equivalent to 1.0 × 10^8^ cfu/mL. The bacterial suspension was evenly spread on the Mueller–Hinton agar plates using sterile swab sticks and allowed to dry. Antibiotic discs (Mast Diagnostics Ltd., UK) with the following drug concentrations were selected for the assay: nalidixic acid (30 µg), ciprofloxacin (5 µg), azithromycin (15 µg), doxycycline (30 µg), erythromycin (15 µg), clarithromycin (15 µg), vancomycin (15 µg), tetracycline (30 µg), clindamycin (2 µg) and metronidazole (15 µg). Plates were incubated at 37 °C under microaerophilic conditions (5% O_2_, 10% CO_2_, 85% N_2_) with gas generator envelopes (CampyGen; 2.5 L Thermo Scientific, UK) for 48 h, and the diameter zones of inhibition were measured, and the results were interpreted in accordance with the CLSI unit [48].

#### Detection of Multidrug Resistance Genes (*cmeA*, *cmeB* and *cmeC*)

Detection of the *cmeA*, *cmeB* and *cmeC* genes was determined by PCR, as described by [49], with slight modifications. Table 2 shows the primer sequences (Inqaba biotech, South Africa). The final concentration of the 25 µL PCR reaction consisted of 12.5 µL of the 2X master mix (Sybrselect, USA) 0.5 µL forward and reverse primers, 6.5 µL molecular grade water and 5 µL template DNA. The cycling conditions were as follows: initial denaturation at 94 °C for 7 min, followed by 94 °C for 1 min, annealing temperatures (see Table 2) for 1.5 min, extension at 72 °C for 3 min and then, final extension at 72 °C for 5 min for 30 cycles. All PCR products were analyzed by electrophoresis on 1% agarose gel (CSL-AG100, Cleaver Scientific Ltd. Warwickshire, UK). The gels were stained with ethidium bromide and visualized with a UV transilluminator and photographed (Alliance 4.7). *Campylobacter jejuni* ATCC 33560 strains were used as the positive control.

## 3. Results

### 3.1. Physicochemical Analyses

The mean water temperature in the Swartkops river estuary for the sampled months was between 14.7 °C and 15.6 °C with the Despatch Mouth (DM) recording the highest mean temperature, while the Rowing Club station (RC) had the lowest mean temperature. No clear-cut difference in pH values was recorded for the sample stations, as the mean pH for all the stations ranged between 8.27 and 8.33. However, a high level of variation in the salinity level was recorded at all stations, with the mean salinity ranging between 13.92 practical salinity units (psu) and 32.77 psu. Station E, which is the dispatch point of Swartkops River to Algoa Bay recorded the highest salinity out of all the sampled months, while the Rowing Club (RC) station recorded the lowest salinity. The average dissolved oxygen (DO) concentrations were 53.83 mg/L and 62.9 mg/L, respectively. Station RC also recorded the lowest DO reading, while the Tiger Bay (TB) station recorded the highest overall reading. In terms of turbidity, the Swartkops water was very turbid during the sampled seasons, ranging, on average, between 4.2 Nephelometric Turbidity Units (NTU) and 66.9 NTU. The Factory Dam (FD) station recorded the highest average turbidity (66.9 NTU), especially in July and August, while DM presented more pristine water for all sample periods. The conductivity was between 20.9 ms/cm and 30 ms/cm on average. A low coefficient of variability was observed for all sampled sites (Table 3).

### 3.2. PCR Confirmation of CJ and Detection of Virulence Genes

**Figure 2:**
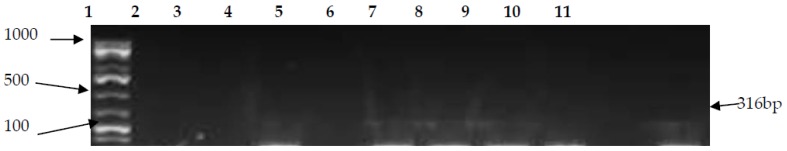
PCR detection of *23S rRNA* gene (316 bp). Lane 1 is themarker, Lanes 2–9 are test isolates, Lane 10 is the negative control, and Lane 11 is the positive control.

**Figure 3:**
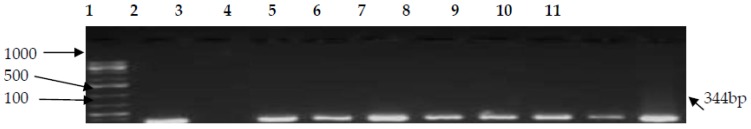
PCR detection of *hipO* gene (344 bp) for *CJ* confirmation. Lane 1 is the marker, Lane 2 is the positive control, Lane 3 is the negative control, and Lanes 4–11 are the test isolates.

One hundred and twenty isolates were phenotypically confirmed as Campylobacteracea (Figure 2). Further screening at the species level confirmed 95 isolates as *C. jejuni* (Figure 3) and the other 25 identified as *C. coli* (18) and *C. upselensis* (7). Determination of the occurrence of virulence genes in the confirmed *CJ* isolates revealed the *cdt*B gene in 80/95 (84.2%) of the isolates, an indication that the toxin production gene (*cdtB*) was the most prevalent virulence determinant. Forty-nine (52%) of the isolates were identified as having adherence virulence genes (*cadF*), while 60/95 (63.2%) isolates tested positive for the intestinal epithelial invasive virulence gene (*cstII*). This gene is also linked to the risk of Gullian–Barre Syndrome (GBS) development. Thirty (31.6%) of the isolates were positive for the lipid A synthesis gene (*htrB*) responsible for the adjustment of organisms to stressful external environmental changes, while 45/95 (47.4%) of the isolates were identified as having the ATP dependent protease gene (*clpP*), which is responsible for the degradation of damaged proteins due to unfavourable conditions. The carbon starvation regulator gene (*csrA*), which is linked to cell division and the formation of biofilm, was absent in all isolates. Isolates recovered from the Redhouse Farm (RF) and Bridge Canal (BC) sampling sites were confirmed as housing all but the *csrA* gene which was absent in all isolates. Table 4 shows the number of genes detected in confirmed *CJ* isolates.

### 3.3. Frequency of CJ Isolation

**Figure 4:**
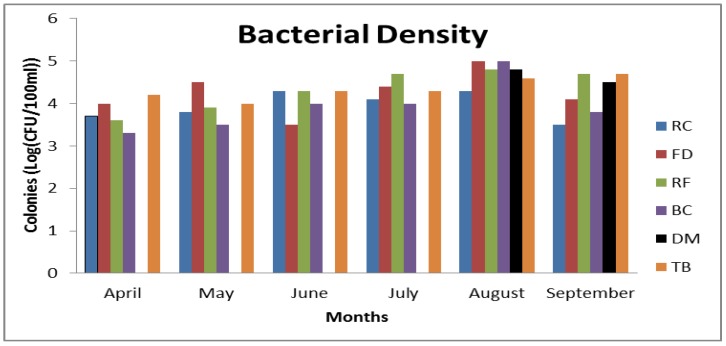
Bacterial density at all sampling sites. * Mean values are expressed in log (cfu per 100 mL).

The frequency of bacterial isolation frequency at all sample sites for the sampling period was recorded (Figure 4). The Despatch Mouth (DM) was the least *Campylobacter*-contaminated site, and the most pristine with an isolation frequency of 33%. No *Campylobacter* count was recorded at DM for April, May, June or July (autumn and winter), but *Campylobacter* were recorded in August and September (spring). The Tiger Bay (TB) site appeared to be the most *Campylobacter*-contaminated site in the Swartkops estuary with an isolation frequency of 100% during the sampled seasons. *Campylobacter* counts were recorded for all sampling months, with higher readings in July, August and September. However, the overall highest average *Campylobacter* counts were recorded at the Factory Dam (FD) and Bridge Canal (BC) sites for the month of August. Consequently, very low counts were recorded in April, May, and June (winter) for all sites.

### 3.4. Physicochemical Parameters and Occurrence of C. jejuni

No significant positive correlation was observed between the population density of *CJ* and temperature (*r* = 0.17), pH (*r* = 0.02), dissolved oxygen (*r* = 0.31), and turbidity (*r* = 0.23). A negative correlation was observed with salinity (*r* = −0.39) and conductivity (*r* = −0.28). The correlation values were not statistically different for temperature, dissolved oxygen, salinity, turbidity and conductivity, while they were statistically different for pH (Table 5).

### 3.5. Antimicrobial Sensitivity Testing and Prevalence of Multidrug Resistance (MDR) Efflux Pump Genes

**Figure 5:**
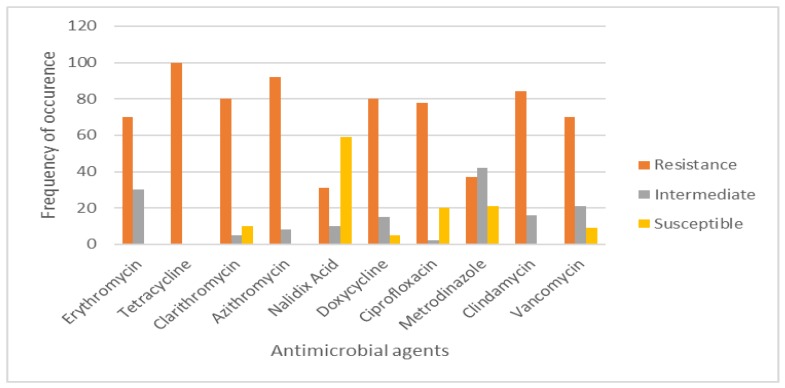
Antimicrobial sensitivity Profile.

The antibiotic sensitivity of 95 *CJ* isolates was profiled and revealed a higher degree of resistance to tested antimicrobial agents. The highest resistance level, 95/95 (100%), was recorded for tetracycline, followed by azithromycin (87/95, 92%), clindamycin (80/95, 84.2%), clarithromycin and doxycycline (76/95, 80%), ciprofloxacin (78/95, 77.8%), vancomycin (67/95, 70.5%), and erythromycin (67/95, 70%), with the lowest resistance levels recorded for metronidazole (35/95, 36.8%) and nalidixic acid (29/95, 30.5%). Nalidixic acid was the most effective antibiotic with a susceptibility of 57/95 (59%) (Figure 5). The percentage incidences of multidrug resistance (MDR) efflux pump genes of *C. jejuni* are shown in Table 6. A high (69/95, 72.6%) number of *CJs* expressed MDR efflux pump genes with only 6/95 (6.3%) not expressing them. Twenty isolates showed a disruption in the expression of all tripartite efflux systems, which could trigger a malfunction of the *cme*ABC system.

## 4. Discussion

### 4.1. Survival of Organism

**Figure 6:**
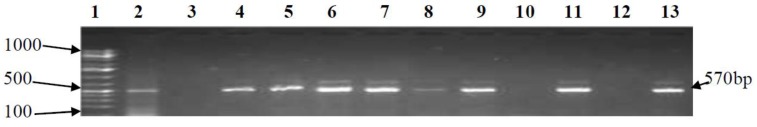
PCR detection of *cstII* gene (570 bp) of *CJ*. Lane 1 is the marker, Lane 2 is the positive control, Lane 3 is the negative control, and Lanes 4–13 are test isolates.

**Figure 7:**
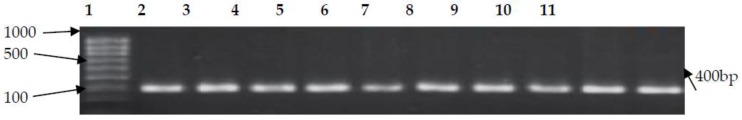
PCR detection of *cadF* gene (400 bp) of *CJ*: Lane 1 is the marker, Lane 2 is the positive control, and Lanes 3–11 are test isolates.

Estuaries are confluent ecosystems where a mixture of salty sea waters and rivers meet with freshwater [42]. They are transition points from land to sea and freshwater to salt water and are rich in organic contents [5]. The dynamic physicochemical nature of the ecosystem is peculiarly detrimental to the survival of fastidious microbes such as *Campylobacter* [14]. However, *Campylobacter* spp. display complex survival mechanisms by transiting to a stationary viable, but non-culturable, form (VBNC) for survival [11]. The recovered strains in this study displayed differential gene expression, which could be peculiarto wild-type *CJ* strains, and morphological evidence showed 66/95 (69%) without flagella. This could be due to the expression of the flagella gene being switched off in a process known as phase variation [49]. The carbon starvation regulator gene (*csrA*) which is linked to the encoding of protein regulation was not detected in the study. The absence of *csrA* could have been complimented by the presence of *htrB* and *clpP* genes. Other associated virulence expressed by this gene is related to oxidative stress survival, adherence of intestinal epithelial cells and in biofilm formation [18,45]. Although the pathogenic potential of wild-type strains is debatable due to the adverse stress conditions which can reduce their colonization and invasive abilities, their ability to live asymptomatically in their hosts may not be fully ruled out, especially as this concerns externally acquired genes from surrounding environs. This is indicated by the presence of the *cstII* gene in some of the strains (Figure 6). Some of the test isolates lacked *cdtB*, and *cadF* genes and could, therefore, be considered non-pathogenic. Most of the isolates lacked motility potential at the point of analysis, inferring the inactivation of the *racR* gene [11]. The results as shown in Figure 7 strongly correlate with the inference drawn in a past study suggesting that *CJ* could still retain its *cadF* adhesion functionality under stressed conditions [14].

**Figure 8:**
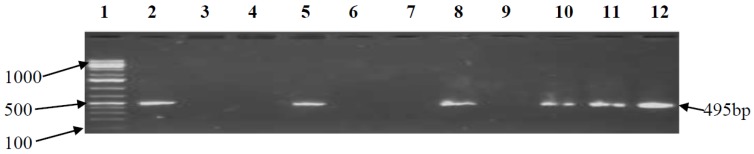
PCR detection of *cdtB* gene (495 bp) of *CJ*. Lane 1 is the marker, Lane 2 is the positive control, Lane 3 is the negative control, and Lanes 4–12 are test isolates.

**Figure 9:**
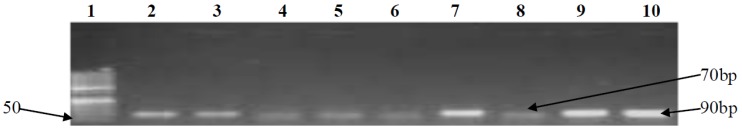
PCR detection of *htrB* (70bp) and *clpP* genes (90bp) of *C. jejuni*. Lane 1 is the marker, Lane 2 is the positive control, Lanes 3, 7, 9 and 10 are *clpP* positive isolates, and Lanes 4, 5, 6 and 8 are *htrB* positive isolates.

The prevalence of the cytotoxin production gene (*cdtB*) in the confirmed isolates (Figure 8) shows that the organism is capable of retaining its toxin production ability even in a starved state. The presence of *htrB* and *clpP* genes (Figure 9) provides an extra boost in the organism’s aero-tolerance survival in environmental waters. However, the survival of microorganisms in oxidative stress environments has been attributed to their ability to develop specialized defensive mechanisms [11]. The existence of *CJ* in the Swartkops could be as a result of oceanic effects rather than continental effects. The FD sample site was the most polluted site in the Swartkops due to effluent discharges from the factory directly to the river. Between FD and BC is the shallowest area; this may be due to high land surface run-off into the river. This area has the highest population of migratory birds and fishing activities with the highest *CJ* density which strongly indicates that avian species are major reservoirs of the organism. Seasonality, on the other hand, could also play a pivotal role in the survival and existence of the organism, as larger population densities of the organism were recorded during spring and the lowest population densities were recorded during winter [2,9]. The Swartkops river estuary has a strong nutrient zonation, which is typified by the variation in salinity distribution in the estuary as a result of upstream shift of salt and fresh waters, which may be responsible for the abundance and distribution of species. Hence, this was the major reason for choosing to investigate the effect of the physicochemical parameters on the survival of the organism. The salinity reading at DM was the highest total average reading of all seasons, but barely affected the survival of the organism.

### 4.2. Drug Resistance

**Figure 10:**
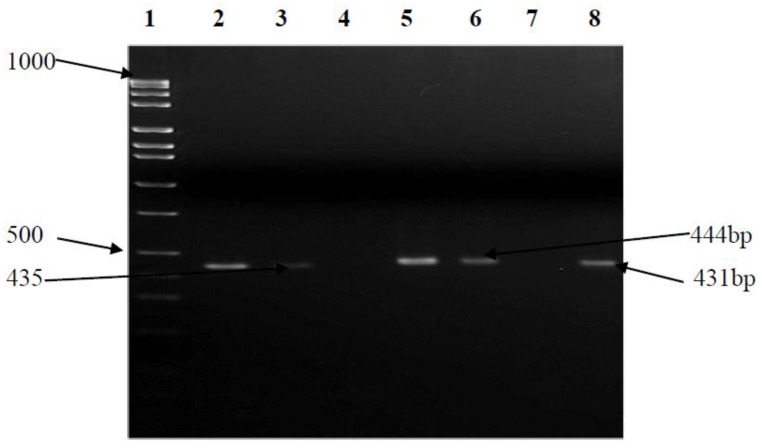
PCR detection of *cme*ABC in isolates. Lane 1 is the marker, Lane 2 is the positive control, Lane 8 is the *CmeA* positive isolate (435 base pair, Lane 4 is the negative control, Lanes 5 and 6 are *cmeB* positive isolates (444 bp), and Lane 3 is the *cmeC* positive isolate (431 bp).

Multiple resistances have been reported globally in both *CJ* and *C. coli* in human and animal isolates. The resistance pattern was observed notably in tetracyclines, macrolides and fluoroquinolones [1,7]. In South Africa, antimicrobials with broad-spectrum activity such as tetracyclines are used in both the poultry and pig industries, as they are affordable and easy to administer in food and water [30]. In a recent study, a high level of resistance to tetracyclines was also revealed in *Campylobacter* spp. isolated from broilers and hens [1]. In this study, multidrug resistance was observed in more than 70% of the isolates (Figure 10). The isolates harbored the *Campylobacter* multidrug efflux pump (*cme*ABC) genes responsible for multidrug resistance [7]. Previous studies have shown variation in the resistance pattern of *CJ* in South Africa [7,30]. Some studies revealed a high resistance of the organism to fluoroquinolones, macrolides and tetracyclines [30], while some showed high susceptibility to fluoroquinolones [1]. Resistance to fluoroquinolone is believed to develop more rapidly in *Campylobacter* spp, than in other Gram-negative bacteria, mainly attributed to single-step point mutations in *gyrA* [2]. However, these studies were carried out on *CJ* isolated from avians, porcines and bovines. In this study, multidrug resistance was shown for fluoroquinolones, macrolides and tetracyclines. A high resistance to tetracycline (100%) was observed and the highest susceptibility (59%) was shown to nalidixic acid. Previous studies have suggested that aquatic environments (surface and groundwater bodies) are perfect for horizontal gene exchange of mobile genetic elements (MGEs) which results in antibiotic resistance [2].

## 5. Conclusions

This is the first study to report the occurrence of differential gene expressions in wild-type *CJ* isolated from the Swartkops in the Eastern Cape Province. The results showed that the estuarine water could potentially harbour multiple resistant *CJ* strains of public health concern among estuarine users. Although the extent of their pathogenicity is not fully ascertained, it could be assumed that pathogens with similar traits are likely to be found in other similar ecosystems.

## 6. Future Direction of Study

This study focused on the detection of active virulence-inducing genes and antimicrobial sensitivity profiling of environmentally recovered *CJ* strains. More elaborate future studies should include a comparative genomic analysis using whole genome sequencing (WGS) for other related water sources of high importance around the Eastern Cape Province to fully understand the pathophysiological mechanisms of recovered wild-type *Campylobacter* strains. Moreover, a comparative study of the antimicrobial profile and analysis of expressed virulence of wild-type isolates and clinical strains should be investigated. Other important virulence determinants which were not investigated in this study such as *rac*R and *flaA* genes associated with motility and *cosR*, *rrpA* or *rrpB* associated with the oxidative stress response should be studied in wild-type isolates. It is also necessary to conduct the same study on other closely-related organisms, such as *Arcobacter* species, to document their pathogenicity.

## Figures and Tables

**Table 1:** PCR primer sizes and annealing temperatures.

Name of Gene	Sequence (5′-3′)	Product (bp)	Annealing Temperature (°C)	References
Campy 23S	F-AATTGATGGGGTTAGCATTAGC R-CAACAATGGCTCATATACAACTGG	316	55	[43]
*hipO*	F-AGAGTTTGATCCTGGCTCAG R-ACGGCTACCTTGTTACGACTT	344	58	[44]
*cdtB*	F-CAC GGT TAA AAT CCC CTG CT R-GCA CTT GGA ATT TGC AAG GC	495	52	[18]
*htrB*	F-CGC ACC CAA TTT GAC ATA GAA R-TTT TTA GAG CGC TTA GCA TTT GTC T	70	52	[45]
*clpP*	F-TCG GAG CAT TTT TGC TTA GTT G R-CTC CAC CTA AAG GTT GAT GAA TCA T	90	52	[46]
*csrA*	F-CAC AGT CAG TGA AGG TGC TT R-ACT CGC ACA ATC GCT ACT TC	878	52	[47]
*cstII*	F-CAG CTT TCT ATT GCC CTT GC R-ACA CAT ATA GAC CCC TGA GG	570	52	[18]
*cadF*	F-TTGAAGGTAATTTAGATATG R-CTAATACCTAAAGTTGAAAC	400	42	[10]

**Table 2:** Primers used in the study.

Target Genes	Primer Sequences 5’-3’	Annealing Temp (°C)	Amplicon Size (bp)
*CmeA*	F-TAGCGGCGTAATAGTAAATAAAC R-ATAAAGAAATCTGCGTAAATAGGA	50	435
*CmeB*	F-AGGCGGTTTTGAAATGTATGTT R-TGTGCCGCTGGGAAAAG	50	444
*CmeC*	F-CAAGTTGGCGCTGTAGGTGAA R-CCCCAATGAAAAATAGGCAGAGTA	52	431

**Table 3:** Physicochemical parameters of water at different sample stations.

Sample Stations	Parameters	Temp (°C)	pH	Salinity (psu)	DO (mg/L)	Turbidity (NTU)	Conductivity (ms/cm)
RC	M	14.7	8.32	13.9	53.8	37.2	20.9
	Cv	0.16	0.02	0.52	0.68	0.85	0.49
FD	M	14.8	8.33	19.2	60.5	66.9	25.7
	Cv	0.16	0.01	0.4	0.67	0.74	0.43
RF	M	14.9	8.27	14.9	55.3	48.4	22
	Cv	0.17	0.01	0.15	0.7	0.78	0.13
BC	M	14.9	8.31	21.4	56.9	31.9	26.3
	Cv	0.15	0.02	0.5	0.69	0.4	0.55
DM	M	15.6	8.29	32.8	58.9	4.2	26.3
	Cv	0.13	0.01	0.19	0.69	0.7	0.23
TB	M	14.9	8.36	25.4	62.9	24.2	30
	Cv	0.15	0.01	0.38	0.68	1.5	0.44

M = mean; Cv = coefficient of variability; DO = dissolved oxygen.

**Table 4:** Detection of pathogenic genes in confirmed *C. jejuni* (*CJ*) isolates.

Sample Source	No. of Samples	No. of Isolates Confirmed as *Campylobacter* Genus for Samples (%)	No. of Isolates Confirmed as *CJ* (%)	Genes Detected in *CJ* Isolates (% Positive)					
Estuarine water	244	23S rRNA	*HipO*	*cdtB*	*cadF*	*cstII*	*csrA*	*htrB*	*ClpP*
		120 (49.2)	95 (79.2)	80 (84.2)	49 (51.6)	60 (63.2)	0 (0)	30 (31.6)	45 (47.4)

**Table 5:** Correlation (Bravais Pearson) matrix between temperature, bacterial contamination, pH, salinity, turbidity, dissolved oxygen and conductivity.

	Temp	PH	Salinity	DO	Turbidity	Conductivity	CJ
Temp	1						
pH	−0.30	1					
Salinity	−0.60	0.46	1				
DO	0.19	0.07	−0.31	1			
Turbidity	−0.08	−0.03	−0.12	−0.43	1		
Conductivity	−0.53	0.48	0.87	−0.32	0.08	1	
*CJ*	0.17	0.02	−0.39	0.21	0.23	−0.28	1

**Table 6:** Incidences of *Campylobacter* multidrug resistance (MDR) efflux pump genes.

Genes	No. of Isolates (*n* = 95)	
	a (%)	b (%)
*cme*A	11/20 (55)	9/20 (45)
*cme*B	18/20 (90)	2/20 (20)
*cme*C	14/20 (70)	7/20 (30)
*cme*ABC	69/95 (72.6)	6/95 (6.3)

a = positive isolates; b = negative isolates.
